# Qualitative evidence of crimes against humanity: the August 2017 attacks on the Rohingya in northern Rakhine State, Myanmar

**DOI:** 10.1186/s13031-019-0227-8

**Published:** 2019-09-16

**Authors:** Nicole Messner, Andrea Woods, Agnes Petty, Parveen K. Parmar, Jennifer Leigh, Ernest Thomas, Douglass Curry, Homer Venters, Andra Gilbert, Tamaryn Nelson, Ed Lester

**Affiliations:** 1 Physicians for Human Rights, New York, NY USA; 2 Clinical Emergency Medicine, University of Southern California, Los Angeles, CA USA; 3 NYU College of Global Public Health, New York, NY USA; 4 South Asian Information Network, Chiang Mai, Thailand

**Keywords:** Rohingya, Myanmar, Burma, Human right assessment, Cross-sectional survey, Mixed-methods, Qualitative

## Abstract

**Background:**

The Rohingya ethnic minority population in northern Rakhine state, Myanmar, have experienced some of the most protracted situations of persecution. Government-led clearance operations in August 2017 were one of many, but notably one of the most devastating, attacks on the population. The study aimed to conduct a multiphase mixed-methods assessment of the prevalence and contexts of violence and mortality across affected hamlets in northern Rakhine State during the August 2017 attacks. This publication describes qualitative accounts by Rohingya community leaders from affected hamlets, with a focus on the events and environment leading up to and surrounding the attacks.

**Methods:**

Qualitative in-depth interviews were conducted with Rohingya community leaders representing 88 northern Rakhine state hamlets across three townships affected by the August 2017 attacks (Maungdaw, n = 34; Buthidaung, n = 42; Rathedaung, n = 12). Prior quantitative surveys conducted among representative hamlet leaders allowed for preliminary screening and identification of interview candidates: interviewees were then selected based on prior reports of 10 or more deaths among Rohingya hamlet community members, mass rape, and/or witness of mass graves in a hamlet or during displacement. Recorded interviews were transcribed, translated, and thematically coded.

**Results:**

Rohingya leaders reported that community members were subjected to systematic civil oppression characterized by severe restrictions on travel, marriage, education, and legal rights, regular denial of citizenship rights, and unsubstantiated accusations of terrorist affiliations in the months prior to August 2017. During the attacks, Rohingya civilians (inclusive of women, men, children, and elderly) reportedly suffered severe, indiscriminate violence perpetrated by Myanmar security forces. Crimes against children and sexual violence were widespread. Bodies of missing civilians were discovered in mass graves and, in some cases, desecrated by armed groups. Myanmar Armed Forces (*Tatmadaw*), consisting of the Army, Navy, and Border Guard Police continued to pursue, assault, and obstruct civilians in flight to Bangladesh.

**Conclusions:**

Qualitative findings corroborate previously published evidence of widespread and systematic violence by the Myanmar security forces against the Rohingya. The accounts describe intentional oppression of Rohingya civilians leading up to the August 2017 attacks and coordinated and targeted persecution of Rohingya by state forces spanning geographic distances, and ultimately provide supporting evidence for investigations of crimes against humanity and acts of genocide.

## Background

After 50 years of military rule, Myanmar held its first democratic elections in 2015, initiating an historic step toward significant political and social reform. Despite this achievement, the shift to a less authoritarian government has not benefited all ethnic groups, including the Rohingya, an ethnic Muslim minority in northern Rakhine state, which borders Bangladesh. A 1982 law denied Rohingya people citizenship in Myanmar [1], and, since then, the Rohingya have faced restrictions on access to education, health care, livelihoods, and on their ability to move freely within the country, even in the case of medical emergencies [2, 3]. The government’s continued enforcement of the law, as seen in the issuance of the National Verification Card (NVC), excludes the Rohingya as one of the 135 national races, effectively labelling them as foreigners [4].

Subsequently, violence against the Rohingya has been ongoing. Beginning in the 1970s, Rohingya have faced waves of violence, often resulting in widespread displacement [5]. Anti-Rohingya propaganda and manipulation of the Buddhist Rakhine population led to Buddhist-Muslim sectarian violence in June 2012 that resulted in the displacement of roughly 75,000 Rohingya [6]. Conflict resumed in October 2016, when Rohingya insurgents attacked Myanmar border posts along the Bangladesh–Myanmar border. The military responded with counterinsurgency tactics targeting the Rohingya civilian population [7, 8].

On August 25, 2017, the insurgent Arakan Rohingya Salvation Army (ARSA) attacked 30 police posts and killed 12 personnel in northern Rakhine state. That same day, members of the Myanmar security forces (military and Border Guard Police officers) initiated a widespread military “clearance” response across northern Rakhine state. Satellite imagery and reports from refugees suggest a coordinated, systematic series of attacks throughout Maungdaw, Buthidaung, and Rathedaung townships, including burning of villages, destruction of property, and violence against civilians. The extreme violence resulted in the death of an estimated 7800 Rohingya [9] and the mass exodus of 723,000 survivors, 40% of whom were under the age of 12 and now live in refugee settlements of Kutupalong and Nayapara in Bangladesh’s Cox’s Bazar District [10].

nvestigations of the attacks in northern Rakhine state have yielded testimonies of male and female survivors utilizing various methodologies [11–13]. Médecins Sans Frontières (MSF) completed a retrospective household survey in refugee camps in Bangladesh which calculated mortality estimates [14], while the United States Department of State conducted a hybrid survey among a large random sample of adults who fled Rakhine state on or after October 1, 2016 [15]. Our research team conducted multiphase mixed-methods research with leaders of affected hamlets to document the scale and scope of the violence, the type of human rights violations, and the mortality rates that resulted from the August 2017 attacks. Our approach is unique in that we capture the experiences of the population from all Rohingya hamlets in northern Rakhine state who had been displaced to Bangladesh following the August 2017 attacks. We included cross-sectional surveys among leaders of affected hamlets [9], clinical evaluations of survivors to medically corroborate their narratives [16]*,* and a qualitative component, as captured in this article. The use of mixed methods is particularly appropriate for studying violence and measuring human rights given the complexity of the subject occurring within a social context [17]. This hybrid, mixed-methods approach aimed to triangulate quantitative and qualitative findings to enhance documentation of the events surrounding the August 2017 attacks. Finally, by interviewing hamlet leaders, we attempted to provide data on entire hamlets, including families or populations who might have been killed and not otherwise represented in the refugee camps. Qualitative findings reported in this manuscript provide contextual details to the quantified experiences of the attacks [9] and physician documentation of associated injuries [16].

## Methods

### Study design and setting

Qualitative interviews were conducted with hamlet leaders residing in refugee settlements in the Ukhiya and Teknaf *upazilas* (subdistricts) of Cox’s Bazar from May through July 2018. Interviews focused on the August 2017 attacks and captured events in affected hamlets from the end of Ramadan (June 24, 2017) to the time when the Rohingya residents of these hamlets arrived in Bangladesh. We developed a comprehensive list of affected hamlets through collaboration with local aid agencies active in the region, compared to the official listing of hamlets from the Myanmar Information Management Unit, and vetted the list with community leaders. This process has been described in detail elsewhere [9].

Ultimately, we identified 604 leaders from affected Rohingya hamlets and urban wards. In Myanmar, the term hamlet is equivalent to village, and multiple hamlets comprise a larger administrative unit called a village tract. Multiple village tracts then make up a township, of which there are three in northern Rakhine state: Maungdaw, Buthidaung, and Rathedaung.

### Participant selection

Six hundred four surveys were completed, representing eight (67%) of 12 urban wards and 590 (>99%) of 591 Rohingya hamlets in the three townships. Two of the 590 hamlets are large and so are administratively split into four subdivisions each, with one representative from each subdivision being interviewed to give 604 respondents in total. One leader from each of the hamlets was invited to participate in this study: the most senior leader present in Bangladesh, or his designee. These leaders included hamlet council chairmen, clerks, secretaries, and village elders. Given the cultural and religious customs of this ethnic group, the positions are held by men; therefore, all participants were male. We focused on collecting hamlet-level information from hamlet leaders for the following reasons: 1) the risk of potential re-traumatization of individuals by household surveys was not ethically justifiable in the context of extremely limited humanitarian resources; 2) household surveys would have extended the time required for data collection and placed data collectors and participants at risk during the impending monsoon season; and 3) extensive reporting requirements prior to the attacks made hamlet leaders experts in the Rohingya populations living in their hamlets and village tracts.

The Myanmar government required (and still requires) that Rohingya hamlet leaders regularly report population data for the Rohingya living in each hamlet for the purposes of facilitating policies that restricted the Rohingya population; thus, local leaders possessed uniquely accurate hamlet-level population estimates prior to flight. Moreover, community members from Rohingya hamlets reconnected after displacement in the refugee camps, and surviving leaders continued to systematically collect and record knowledge of the Rohingya population of their affected hamlets. Community consultations conducted prior to data collection corroborated that collecting information from hamlet leaders was ethically justifiable, and would yield accurate reports and mortality estimates associated with the events surrounding their hamlets before, during, and after flight to Bangladesh. Indeed, numbers of displaced persons and mortality estimates calculated through this approach [9] were similar to those produced through other household surveys.

A cross-sectional survey was conducted among all identified leaders of the affected hamlets to capture hamlet-level quantitative data documenting events leading up to attacks; types, locations, and perpetrators of violence; and experiences in displacement within Rakhine state and to Bangladesh [9]. Any survey participant who reported 10 or more deaths of villagers, mass rape, and/or witnessing of mass graves in their hamlet or during displacement was invited to participate in a qualitative, in-depth interview [9]. This selection was programmed into the electronic survey tool that automatically identified participants who fit the criteria based on their survey responses.

### Data collection and analysis

In-depth interviews were conducted in private on the same day as the survey interview, unless the participant requested to reschedule. Qualitative participants were asked to elaborate on and provide a detailed description of events captured in the survey. Interviews followed a semi-structured interview guide that included open-ended questions on the following topics: military build-up and community meetings held prior to the attacks, events during the attacks, dates of events, reasons for flight, events during flight, experiences living in the camps in Bangladesh, whether or not they would consider returning to Myanmar and under what circumstances, and hopes for the future.

Interviews were conducted by Rohingya data collectors trained to translate from the written English interview guide directly into Rohingya, which is an oral language that lacks a written form. Interviews were audio recorded and recordings were transferred to password-protected computers and removed from recording devices. Recordings were transcribed and translated by individuals who had demonstrated the accuracy of their translations to the research team. English transcripts underwent qualitative analysis using content analysis strategies based on the domains broadly outlined in the topics stated above. Qualitative coding was performed by two analysts from the research team using Atlas.ti (Cleverbridge, Inc., Chicago, IL) and supported by ongoing discussions with the wider team.

### Ethics approval and consent

Ethical approval for all components of this mixed-methods investigation was obtained from the Ethics Review Board of Physicians for Human Rights (PHR). Given the absence of a formal Rohingya body that could serve as a review board, a community consultation was held with Rohingya community leaders in the Bangladesh camps prior to implementation to obtain their input, feedback, and approval. The consultation included *majhis*, block leaders, teachers, imams, former Village Council chairmen, and members of the Arakhan Rohingya Society for Peace and Human Rights. The risks of participation were discussed with the survey team and with community leadership during the community consultations. Surveyors were trained to recognize signs of distress, manage respondents experiencing distress, and facilitate appropriate referrals to medical care or psychosocial support, if needed. Both groups concluded that the plans to mitigate these risks were adequate and endorsed the study.

It was of utmost importance to protect their identities and to prevent possible retaliation if and when Rohingya are able to return to Rakhine state. To protect the safety of participants, no names or identifying information beyond hamlet and title were recorded during data collection; qualitative data presented in this manuscript are presented at the township level to prevent potential identification of hamlet leaders.

All participants gave full and informed verbal consent prior to participating in this study. A separate consent process was conducted for participation in the qualitative interview following the quantitative survey; this consent included additional consent to audio record the interviews.

## Results

Nearly one quarter (23%; 139/604) of survey participants met the identified threshold for in-depth interviews. However, interviews were discontinued after 88 were conducted across Maungdaw (34), Buthidaung (42), and Rathedaung (12) townships, given that sufficient information had been collected to achieve data saturation [19] across a wide geographical area and due to resource constraints related to costs of translation and transcription of interviews.

### Human rights violations prior to August 2017 attacks

#### Targeted discrimination

The qualitative data analysis highlighted a number of reoccurring themes, the first being the rapid restriction of the Rohingyas’ legal and civil rights. Qualitative participants repeatedly spoke of the changes they witnessed over the last two decades, most notably following the events of June 2012 and October 2016, and the more abrupt and intensified changes in the months leading to the attacks of August 25, 2017. With each event, human rights violations, including the numerous restrictions faced in daily life, increased significantly. Interviewees shared memories of once living together peacefully, but now described their life as being “trapped.”

Life was so miserable. First, they demolished the fences of our houses. They restricted our movement from one village to another. We needed to pay 1000 kyats (USD $0.70) for approval to pass to another village. No Rohingya student was allowed to go to Sittwe University. Extortion and arbitrary arrest happened on a daily basis.… Many died due to lack of timely medical assistance. Pregnant women would die on the way due to delays in obtaining approval. We were prevented from getting married. Newborn babies were excluded from the official birth records on family lists. Life was an open prison. (Buthidaung #26)

The spectrum of restrictions varied between villages, but Rohingya reported that their lives were more restricted than those of their non-Rohingya neighbors. Local officials required Rohingya to pay fees to marry or have children and limited family size.

Some people get permission [to marry], whereas some do not. In the marriage permission law, there is a condition that only two children can be born. If more than two children, the parents were imprisoned and money extorted. (Buthidaung #12)

Many also said they were unable to practice their religion, because the military closed religious schools, prohibited mosques from issuing a call to prayer, or, in some cases, burned down the mosques.

They didn’t allow us to rehabilitate the old and broken mosques, and many people were imprisoned because of building mosques.… All the main mosques and Arabic schools have been closed since 2012. (Maungdaw #34)

Restrictions on movement for the Rohingya were particularly severe, with a complex web of regulations making it extremely difficult for Rohingya to move freely. A curfew imposed at night prohibited people from leaving their homes while local law enforcement kept strict watch at street corners, which was presumed to be for the purposes of arbitrary detention of Rohingya residents. Exceptions for health emergencies were not granted, as described above. One interviewee shared: *“Once, the doctor recommended that my son go to Sittwe Hospital to receive a specific treatment. But I was not allowed to go. Like me, there were many prevented from going to the hospital.” (Buthidaung #3).*

#### Mandatory meetings with government officials

Rohingya reported notable changes to life in their hamlets during the 3 to 4 months leading to the events of August 25, 2017. Every qualitative participant interviewed provided details of regular meetings that were convened either at the village tract or hamlet level by military personnel and/or members of the Border Guard Police (BGP) and Immigration Department. In many cases, only Rohingya leaders or administrators were summoned, frequently at gunpoint, to attend the meetings. The number of meetings ranged from one to as many as 15. In some hamlets, the commanding BGP officer called every resident to attend and threatened to shoot anyone who was absent from the meeting.

Interview data reveal two recurring threats made by the military and BGP officers and the main reason for these meetings: 1) accept the NVC and declare oneself “Bengali” (a pejorative word in Myanmar whereby the respondent would be renouncing “Rohingya” ethnicity and self-recognizing as not having Myanmar citizenship); and 2) identify individuals in one’s hamlet who are members of ARSA. A 50-year-old hamlet headman from Buthidaung describes the following:

They said in the meetings that we must hold the NVC if we wanted to live in Myanmar. When we denied the NVC card, they wouldn’t let us travel anywhere. Sometimes we had to go hungry as we couldn’t go anywhere to buy food. In the meeting, they also told us that we are not Rohingya, we are Bengali. We have to obey everything they say. They also threatened us in the meeting that they would kill all men between 12 and 50 years old. (Buthidaung #16)

Qualitative participants repeatedly expressed confusion over why they would accept a card that labels them as outsiders when they and their ancestors have shared the same land for generations and were previously recognized as citizens by the Myanmar government. *“We deserve the same rights like others in Myanmar.” (Maungdaw #41)* In Rathedaung Township, one qualitative participant emphasized how the intentions of the security forces were not to force them to accept the NVC, but rather to systematically eliminate the Rohingya ethnic group.

At the meeting, the most senior officer, the Battalion Commander, said “We have been sent here from Burma to kill, to eliminate you, Bengalis. We have been sent here to attack Bengalis, to burn the houses of Bengalis. You must leave as early as possible. Otherwise, we will keep on burning down (your villages) one after one. We will drive you out by shooting and by attacking you with launchers. You must all leave.” (Rathedaung #5)

#### Arrest and detention

The drive to find ARSA members also fueled violence during meetings. Many qualitative participants spoke of the police and military’s hunt for “bad people” and the need to arrest or kill those individuals. Some Rohingya were confused by this notion of “bad people” and didn’t make the connection between these targets and ARSA. Others expressed frustration that they were required to name ARSA members, but, in honesty, did not have this information. “*They said to point out the bad people. To whom should we point out as bad people without knowing them?” (Maungdaw #14)* If no one was identified, the Myanmar security forces often arrested the wealthiest and most educated village tract residents. A village administrator explained, *“People who are rich and wealthy were not allowed to stay there. They were exiled first. Then, the educated people were targeted and exiled. (Maungdaw #19)”* Muslim leaders known as “mullahs” were a frequent target for arrest and deportation. News spread quickly that these individuals were at risk. Many of them, though reluctant to leave Myanmar, reportedly fled to Bangladesh in the months prior to the August 2017 attacks.

Table 1 describes the demands and threats made during meetings with Myanmar officials.

**Table 1:** Reported threats by Myanmar government representatives against Rohingya prior to August 2017

Threats	Exemplary Quote
Physical violence and forced eviction	*In the meeting, they said there were bad people in your hamlet. “You have relations with them. If you did not find them, we will shoot you, torture you and drive you from the hamlet.” (Maungdaw #26)*
	*We have to arrest the bad people. If we can’t, they would come and burn out the whole village. (Maungdaw #2)*
Destruction of Rohingya village	*If we reject NVC, military and BGP threatened us they would make our village the land of ash. (Buthidaung #5)*
	*And they threatened us they would make our village burnt to the ground. (Maungdaw #21)*
Loss of land and property	*We would have “to receive NVC; if not, we would not be allowed to stay in the land and they would bring Buddhists from other areas to ours.” (Maungdaw #7)*
	*In the meeting, they told that they would make us landless, helpless, and property-less (Maungdaw #10)*
Loss of livelihood	*They said “You will not be allowed to fish. You will not be allowed to go anywhere. You will not be allowed to do any business. You will not be allowed to go to the mountain. You will not be allowed to do anything. We will make you die from starvation.” (Buthidaung #24)*
Forced eviction	*They said in the meeting that we could not stay if we didn’t obey everything the government said. Also, they said that there was “nothing we owned; all belongs to the government.” (Maungdaw #21)*
	*“If you want to live in this country, you have to follow instructions. If you don’t follow our instructions, for us, to kill you is as easy as killing ants and dogs, as you are Bengalis, the illegal immigrants.” (Rathedaung #7)*
Retaliation	*The commander of [name omitted] attended and told us that if any Buddhist was killed, they would kill a thousand Rohingyas. (Maungdaw #11)*
	*They always threatened us in advance that they would “burn the whole village, destroy it by bulldozer, and kill all the people if any violence occurred.” (Maungdaw #11)*

#### Attacks of August 2017

Each qualitative participant provided remarkably similar details of what he and other villagers experienced on and around August 25, 2017 and how they came to flee to Bangladesh. Most begin at night with the eruption of barking dogs or the distant buzzing of machine guns from neighboring hamlets. Military trucks or men on foot abruptly appeared, eliciting instant fear and chaos. People instinctively ran out of their homes and into the surrounding fields or woods. Some people ran away to the nearest neighboring hamlet or dense area of jungle, while others navigated their way to the river and swam. The gunfire continued intermittently throughout the night. At sunrise, the perpetrators entered the hamlet and began setting each house on fire.

Respondents described villagers being shot, tied to burning buildings, and/or raped during these attacks. The most vulnerable, the elderly and children, were reportedly lifted into the fires or dismembered and thrown into ponds or other pits. One survivor, a 42-year-old village administrator from Maungdaw Township, shared his experience.

When they started the horrible violence, all the villagers went to the nearest sandy river shore where there was no other way to run because that place was surrounded by river banks and BGP camps. Then, they heartlessly beat all the people by machine guns. Some women who were holding their crying babies were forced to get into the river and the babies were thrown alive into the river. After that, the women were killed. Around 100 young girls and women were taken back to the village. After some time, one of the few remaining houses was burnt down, then the next one, until they all were burnt down.… The military dug a mass grave and collected the dead bodies and made three piles. Some who had drown[ed] were taken by the water flow. Other military men were slaughtering and beating with a strong stick the badly injured people….Only 10 half-burnt and injured women survived from all the women taken back to the hamlet. They arrived in Bangladesh, and they are still staying here. Some have head injuries, some are without hands or legs. If we see them in the camp, we can’t control ourselves and feel ill. (Maungdaw #24)

#### Mass graves and executions

The high death rate associated with the August 2017 attacks, coupled with the tactics used to kill groups of Rohingya, gave rise to mass graves in all three townships. The manner in which the perpetrators handled the bodies, as described by one hamlet leader in Maungdaw, also demonstrated the violence and lack of dignity with which the Rohingya were treated.

Six kids were shot to death and put in a mass grave. After shooting them, the military sent their dogs to bite them. Soon, the military slit their throats and cut off their genitals and took out their eyes. It was heart-stopping seeing that. (Maungdaw #7)

Qualitative participants from Buthidaung township witnessed Myanmar security forces blindfolding groups of villagers and leading them away, presumably to be killed. One hamlet leader detailed how the police wrapped clothing around the heads of men, women, and children then forced them at gunpoint to a remote setting outside of the hamlet. The following day, he returned to the area and saw streaks of blood along a dirt path which eventually led to a pile of bodies at a mass grave.

When we came back to the hamlet to see the situation, we saw seven mass graves close to the compound at the northern side of the hamlet, three graves in a rice paddy field east of the main road, and one grave in another place in the cemetery. Each grave was around 10 square feet and 15 feet deep. We estimate 50 bodies in some graves. Some people were thrown into a big pond – around 50 dead bodies. Some bodies had their arms cut off, some [were] without legs and some were headless. The rice mill and market were already burnt down. The military brought acid from the market and put it on the mass graves. (Buthidaung #7)

In some cases, the mass graves were not discovered until the villagers began their flight to Bangladesh:

While I was walking, I often looked back at our village. My heart did not want to leave but the situation was miserable. Soon, I saw the dark green rice plant in the field of [hamlet name omitted]. I went there and found the mass grave. Many dead bodies. Faces were burnt and smelling very badly. I lost my mind seeing them. It is south of the market in [hamlet name omitted]. There could be hundreds of dead in the field. (Buthidaung #25)

#### Mass rape and sexual violence

Hamlet leaders described contexts in which groups of women were taken to a nearby building and sexually assaulted. They often were kept for the entire night and released in the morning. The next day, the perpetrators would come and take another group and return them at daybreak. Girls, some as young as 8 years old, were raped in front of their parents or siblings.

*While women were being raped, some men tried to save them as they couldn’t watch this happen to their mothers, daughters, and sisters.* (*Maungdaw #10).*

The individuals who were assulted suffered a range of physical injuries including death.

*Eight teenage girls were taken to another place, where they were very brutally raped and even had their female organs cut. From this group, three girls were killed. When a father tried to save his child, they shot him.* (*Maungdaw #34)*

The hamlet leaders reported that some women in their village chose not to share their experience of sexual violence. One qualitative participant described the challenge of estimating the number of people among the Rohingya population who had been raped.

*Yes, the military committed rape on so many women and girls but the women are ashamed to express this. They are afraid to speak out against the military. So instead they turn to God and keep silent.* (*Maungdaw #2)*

Patterns of rape and sexual violence were reported prior to the attacks of August 2017 and increased as authorities made more frequent visits to the villages to hold mandatory meetings. They consistently revealed accounts of sexual harassment, forced nudity, and gang rape during routine village “spot checks” made by the police and military.

#### Children specifically targeted

Prior to the August 2017 attacks, child abuse by the BGP was reportedly common when Myanmar security forces routinely entered villages to threaten and terrorize residents. Young Rohingyas, hearing their arrival, would frequently run to escape and hide, though anyone seen running would be a target for gunfire. Many qualitative participants described how children feared leaving their house or traveling to school.

Children were not going to school, because the military walked the streets all the time. They used to beat and torture Rohingya children if they found them on the streets. They usually beat children and would throw them in the pools nearby the streets. (Rathedaung #11)

In Maungdaw Township, several participants explained how *“children were also tortured to ask about bad people.”(Maungdaw #28)* Adults witnessed the police beating and kicking young boys and girls to solicit information on ARSA members.

During the August 2017 attacks, Myanmar security forces reportedly made little distinction between adults and children as they entered each village; violence was experienced by adults and children alike. Many children were shot and many who did not die from the gunshot wound had their throats slit. Informants witnessed the perpetrators picking up and throwing children into burning houses. Others were thrown into water ponds and into pits dug for mass graves*.*

*When they found some children alone, separated from the parents, they slaughtered them and cut them into two pieces. (Buthidaung #18).*

#### Myanmar security forces

Qualitative participants identified BGP, the Myanmar military, and civilians, including non-Rohingya neighbors in Rakhine state, as perpetrators of the ongoing violence in the years leading to the attacks of August 2017, as well as the attacks themselves. Many agreed that the Rakhine knew of the government’s plan to drive the Rohingya out. One village leader recalled a Rakhine neighbor joke: *“Why are you farming so hard? You can’t eat these! You will have to leave it.”* (Maungdaw #28) Ethnic Rakhine neighbors entered Rohingya villages and stole vegetables from the gardens, livestock from the fields, and fish from the ponds. “*When we complained to the police and local authorities, they ignored it. So then, we came to know it is well coordinated.” (Maungdaw #29).*

Many participants reported that during the August 2017 attacks the Myanmar military utilized machine guns, rocket launchers, and landmines as weapons for their ground assault.

They set up land mines in the fields outside all the Rohingya villages…they took positions on the side of the road…they started to shoot with launchers inside the village and set fire to the houses. Then people ran out of the houses. When people ran out of the village and reached the field outside, around 100 people died in the field. (Buthidaung #9)

In addition to attacks from the ground, participants described gunfire from helicopters.

Soon, the helicopter was flying above. People were trying to hide and entered into the mosques. The helicopter started firing at people. Some say there were two helicopters attacking the villagers. I was hiding inside the mosque and panicked hearing the sound of firing from helicopters. People were scattered and running here and there. During the late afternoon, the helicopters went away and the firing stopped. Soon, two speedboats arrived to [hamlet name omitted] village and the helicopters landed. From a distance, I saw men in military uniforms in the speedboats. (Maungdaw #3)

#### Flight from Myanmar

Interviewees reported that many survivors escaped because they hid in the forest, undetected by the perpetrators. Some were reported to have waited through the night while others went without food for days before returning to their home and witnessing the destruction. The majority of qualitative participants expressed their determination to stay in Myanmar and wait out this crisis, because *“no one wants to leave the motherland.”* Other residents fled to neighboring hamlets; however, they found that this was not a safe option, as many hamlets experienced the same violence.

We moved from one village to another to find a safe place to stay…just to save our own lives, just to avoid being killed, we continued to look for a safe place and reached Maungdaw township. Here we found dead bodies everywhere. All the villages had already been burned down, and there were dead bodies inside the houses in the villages. It looked like a battlefield. (Buthidaung #23)

The chaos of the attacks separated families and hamlet residents. Many people were forced to begin their journey to Bangladesh without locating their parents, spouses, or children. Because people fled to nearby hamlets, and at different times, new groups formed to make the trek to Bangladesh. If the crowd was too large, one strategy was to reorganize into smaller, more manageable groups, potentially to avoid detection. Some consisted of only 10 or so Rohingya, while others included 200 or more. As they approached the border, they sometimes merged into groups of 20,000 or 30,000 people.

#### Vital events: births and deaths in transit to Bangladesh

Along the way, qualitative participants reported that the Rohingya people experienced multiple challenges to reach Bangladesh safely. Between August and September 2017, a total of 63 in. (1.6 m) of rain hit northern Rakhine state [20]. The heavy rain in the forest weighted survivors’ clothing, made roads and upward paths difficult to climb, raised the level of the river beds, and exposed them to an increased number of insect bites and to mosquito-borne illnesses; these challenging conditions likely contributed to a large number of non-violent deaths.

Some drowned in the water while crossing the creeks. We passed the nights in open fields with women and children together without shelter. Insects bit us…We starved for days. (Maungdaw #19)

For many, the route entailed crossing three mountain ranges such as Lobboi Dhala, which was known to be laced with landmines. One group journeyed with a single cow who took the lead and walked first along the path to identify landmines. Another group chose four “front-runners,” who would walk ahead and signal if military and BGP were sighted.

Displaced villagers reportedly became weak from lack of food, consuming only leaves and herbs, and some suffered from diarrheal illnesses and fevers. Men commonly had to carry elderly relatives on their shoulders. Some of these older individuals were left along the way, as they were too weak and could not walk further. *“It was an unforgettable voyage in life,”* said one hamlet leader from Maungdaw Township.

Another man from Buthidaung shared:

Wherever we went, we saw half-burnt hamlets, crossed dead bodies, and saw mass graves in which many people were burnt and buried. To protect our frightened children, we put clothes on their eyes. While we were travelling through the forest avoiding military men, we saw old people who had been left behind by family members. Most groups, including ours, faced a shortage of food. We ourselves buried four dead bodies in one grave on the way, although it was difficult to perform Salat al-Janazah (final prayer). (Buthidaung #15)

Qualitative findings also revealed that across the townships women gave birth during the flight to Bangladesh. Most reports from qualitative participants of these deliveries included the death of the baby and, sometimes, the mother.

There were deliveries. A woman delivered a baby on the peak of the mountain. There was no doctor. Soon, the baby was dead, and all were crying. The family had others to carry over the mountain, so the mother was left there in the mountain. (Maungdaw #13)

In some cases, the baby received timely medical attention, if close to the Bangladesh border. “*There were two deliveries on the way. One baby was dead because of lack of medical assistance and another was rescued by the support of Bangladesh’s BGP force.” (Maungdaw #9).*

Qualitative participants noted that many of these women who gave birth were delivering pre-term. One participant provided details of his experience witnessing a woman he knew give birth during the flight to Bangladesh. As they rested along the river embankment, several men took a stretcher off of their shoulders that was being used to carry an elderly man. Suddenly, the stretcher was used by a mother to give birth to a premature baby. He believed that the “*weariness”* of the journey induced early labor.

#### Ongoing attacks during flight to Bangladesh

During flight, the Myanmar security forces and BGP reportedly blocked the main roads, requiring the displaced Rohingya villagers to use more hazardous routes. Many groups assigned scouts to assess for signs of security forces and would change direction if necessary. Some experienced direct gunfire along the way, but the majority reported that they did not encounter security forces again until they reached the river at the border crossing.

Fishing boats, often overloaded with people, made attempts to cross the Naf River into Bangladesh. Amid the thousands of families waiting to cross, those who still had money by the end of their journey were permitted to board first.

We finally reached to border. Here we found thousands of people waiting to cross the river. Those who could afford to pay for boats were crossing and those who could not were waiting in desperation. We took a boat and had to pay 80,000 takas (USD$ 100). (Maungdaw #7)

Qualitative participants reported that Myanmar’s navy pursued boats transporting people across the water, while other military forces stayed onshore. Armed forces continued their assault with gunfire, claiming the lives of people both in the boats and waiting on the river bank.

The military didn’t allow us to cross the river when we eventually got to the border. While some people were crossing by rowboat, the military shot and killed five people on the river. Among them, one is a very famous religious mullah leader (name omitted). Bangladeshi BGP and people brought the dead bodies and buried in Bangladesh. (Buthidaung #2)

#### Looking forward

Qualitative participants described their lives as refugees in crowded camps with small temporary shelters. Despite the scarce resources, they shared their gratitude towards Bangladesh and the international and local agencies caring for them. When asked about what they require today, their answers reached beyond their immediate needs in the refugee camp. They recognized the recurring routine of violence and flight and shared their desires for a peaceful life back in Myanmar, commenting: “*We breathed the violence, convincing our heart that that is our country and nothing lasts foreve*r.” *(Maungdaw #38)* They found strength and comfort in wishing and hoping for a better future.

For decades, we have fled and returned to Myanmar many times. How long should we continue to flee? Were we born just to flee and return? This time should be the last ever. The international community should solve this crisis standing firmly with us. Without the assurance of safety and citizenship for us in Myanmar, going back is to face death and flee the same thing again, and there would be the end of our lives. We no longer want this pain and persecution. Our heart trembles to feel once the full freedom. (Maungdaw #38)

Thematic analyses identified four recurring requests when asked about the future: 1) full restoration of Rohingyas’ legal and civil rights; 2) protection and security provided by the international community if repatriated; 3) reparations for property confiscated or destroyed; and 4) access to adequate education for their children. Table 2 includes supporting testimony for each main request.

**Table 2:** Most frequent requests for the future by Rohingya refugees and supporting quotations

REQUEST	EXPLANATORY QUOTE
Equal rights	*We need for our people to be able to live and do all the activities freely in the same way as Rakhines and Burmese are able to do.* *(Buthidaung #23)*
	*We need to get a citizenship card with the recognition of the ethnic name “Rohingya.” (Rathedaung #9)*
	*Rohingyas must be allowed to serve as military, police, BGP, and all other government services. Rohingyas must have a chance to become government officers in the same way as Rakhines, Burmese, and other ethnic groups. (Buthidaung #8)*
Reparations	*We must get back all our properties, such as the land confiscated by military battalions and other Buddhist ethnic groups, and our cattle seized by them. We must get compensation for all these.* *(Buthidaung #9)*
	*We must get compensation for all the properties we lost. For example, I used to have 10–20 cows and goats. I used to have a big strong house. I lost all these properties of mine. (Buthidaung #17)*
	*We had to leave behind all our properties that we earned for our whole life and inherited from our parents. [The] government must pay compensation for all our properties that we had to leave behind.* *(Buthidang #24)*
Education	*The most important need is for our children to be able to seek education in the same way as Rakhines and Burmese children.* *(Buthidaung #23)*
	*If our children cannot get education here either, when we return to our country, we will still be excluded from all roles, saying “you are not educated.” That’s why we worry about our children’s education.* *(Rathedaung #13)*
	*After arriving here in Bangladesh, our main concern here is that our children will become illiterate; our children will lose knowledge… How can we give our children an education?* *(Rathedaung #4)*
Security	*The international community should stand firmly with us until we have our own rights in our country, so we can live up there in safety and dignity like others.* *(Maungdaw #7)*
	*They would receive us and kill us again without the insurance of safety and security for our lives.* *(Maungdaw #22)*
	*To live peacefully there, we really need the security forces who can protect our lives and properties.* *(Maungdaw #19)*

Qualitative participants expressed that restoring basic rights requires the government to recognize the Rohingya as one of the official national ethnicities in Myanmar. Participants indicated that citizenship status should be accompanied by a lift in restrictions and protection to engage in daily life without violence and persecution. Many recalled experiences from older generations who fled Myanmar as refugees. When they returned, the government broke their promise and continued to abuse them. “*They did not grant them any rights. This time, if you can get us our rights truly and correctly, we will return right away. We are very willing to return to our country. If not, we will not leave Bangladesh.” (Buthidaung #30).*

When considering repatriation, participants pleaded for protection against the authorities who attacked them. They described their distrust of the government and fear that, without the backing of some form of impartial peacekeeping security forces, their lives will be in danger once again, which demonstrates the fragility of a possible return. *“If they are with us, we will feel safe and protected.” (Buthidaung #29)* Additionally, repatriation raised questions among participants in terms of how to rebuild. “*Where will we live when we reach back home?” (Buthidaung #29)* As refugees, many express that they have limited personal possessions and income and are dependent on external aid to secure land, homes, and livestock, and to slowly create a viable living environment.

Another common and repeated request was to enroll children in school to ensure they have full access to education. “*For us, education for our children is more important than receiving food aid.” (Buthidaung #6).* Participants criticized the quality of educational provision in the camps, describing the lack of formal instruction and the exclusion of adolescents. They uniformly indicated access to education as a priority and understood the ramifications of an under-educated youth population. “*In this world, there is no way to live well without proper education.” (Buthidaung #30).*

## Discussion

Myanmar’s armed forces claimed responsibility for the fighting in August 2017, but the government maintains that their actions did not violate the human rights of the Rohingya [12, 21]. Instead, they describe these attacks as a legitimate counterinsurgency operation designed to target ARSA following the attacks earlier that month [12]. Evidence from our qualitative interviews described here, as well as our quantitative findings [9] and physician-investigator physical exam documentation, corroborate other reports [11, 12, 15, 22, 23] and indicate that the attacks were part of a coordinated, ongoing military campaign to expel and eliminate the Rohingya community.

Findings reported here provide context into how the violence unfolded, with a specific focus on descriptions of mass graves and executions, mass rape and sexual violence, as well as the impact on specific populations affected, such as children. It also gives details on the role of Myanmar security forces before, during, and following the attacks, as hundreds of thousands of Rohingya fled to Bangladesh on a treacherous journey across great distances and under continued attack.

The ongoing state policy restrictions described by qualitative participants reportedly deteriorated the quality of life for the Rohingya so much so that many left their homes prior to the attacks. Between 2012 and May 2017, UNHCR estimates that approximately 168,000 Rohingya had already fled to either Malaysia, India, or Bangladesh [24]. Hamlet leaders participating in this research indicated that those who stayed endured a steady increase of military and police presence. Over 90% of affected hamlets that participated in our quantitative survey reported forced meetings with authorities in the time leading up to August 2017 [9]. Moreover, hamlet leaders explained how the wealthy and educated Rohingya often were the first to be arrested and never return. The UN identified this strategy as part of a deliberate plan to destroy community resources and to debilitate the Rohingya population’s ability to ever rebuild itself and function in Myanmar [22].

Results of our quantitative survey around the attacks of August 2017 demonstrate the widespread and systematic nature of the violence: for example, 89% of hamlet leaders reported violence directly against their hamlet, while 64% also reported violence during flight, including with the use of mortars, artillery weapons, and other heavy-fire machinery [15]. The widespread and systematic nature of these attacks are further supported by this qualitative research and associated physical exams, which document the extent and brutality of violence perpetrated in hamlets spanning three townships, coordinated attacks by multiple armed forces with heavy military assets, the number of deaths and mass graves in hamlets, attacks during flight, and desecration of human remains (Table 3).

**Table 3:** Relevant crimes committed prior to and during the August 2017 attacks on Rohingya communities of Rakhine state: mixed-methods documentation, including testimony from this qualitative research and results from physical exams [16] and quantitative survey [9]

CRIME	SUPPORTING QUALITATIVE QUOTES	SURVEY RESULTS [9]	PHYSICAL EXAM RESULTS [16]
*Genocide:* any of the following acts committed with intent to destroy, in whole or in part, a national, ethnical, racial, or religious group			
(a) Killing members of the group;	*While fleeing, we saw pits full of people (dead bodies) and there was no way to count.* *(Buthidaung #8)* *Some women who were holding their crying babies were forced to get into the river and then the babies were thrown alive into the river. (Maungdaw #24)*	7803 Rohingya died from violent and non-violent causes associated with the August, 2017 attacks and subsequent displacement.	(All physical exams conducted among survivors).
(b) Causing serious bodily or mental harm to members of the group;	*We can’t do anything or work by our physical structure because of their brutal torturing.* *(Buthidaung #16)*	89% of hamlets reported violence in their hamlets before flight, most commonly including injuries with weapons, starvation, and attacks on religious leaders. 64% reported violence against civilians in flight, most commonly including shootings and the use of landmines and other weaponry.	Gunshot wounds were clinically verified among 68% of physical exam participants; permanent disability among 41%; injury from fire/explosion among 40%; blunt force trauma among 22%; and psychological trauma among 20%.
(c) Deliberately inflicting on the group conditions of life calculated to bring about its physical destruction in whole or in part;	*We came to understand that even after destroying all our villages and our properties, even after killing people while running away to escape from the attack, they still intended to kill more people. There was no way to survive inside Myanmar. They would eliminate all of us – even our young generation. (Rathedaung #9)*	94% of hamlets reported that their hamlet experienced destruction, including burning or destruction of fields or farms (84%), homes (80%), and mosques (69%). In addition, residents experienced restrictions to travel, marriage, and other rights.	Survivor testimonies recounted the organization and execution of the attacks on the Rohingya. Survivor testimony noted that both Muslim and Buddhist health care providers refused care to Rohingya for fear of being persecuted or targeted themselves. After the attacks in August 2017, some respondents described attempting to reach local health workers to treat their wounds but being turned away. Physician-investigator assessments determined that many of these delays resulted in permanent disabilities.
(d) Imposing measures intended to prevent births within the group;	(No data identified)* *Measures to prevent marriages was documented: *Authorities restricted marriages. They just postponed it by finding excuses such as “people getting married haven’t reached legal age; the data on the documents submitted is wrong,” and so on. Finding excuses, or faults, they restricted, for example, 10 marriages out of 15 marriages of the applications submitted. We believe that their purpose was to decrease our population.* *(Buthidaung #34)*	(Not measured)	(Not reported)
(e) Forcibly transferring children of the group to another group.	(No data identified)	(Not measured beyond attacks against and murder of children)	(Not reported beyond attacks against and murder of children)
*Crimes against humanity*: any of the following acts when committed as part of a widespread or systematic attack directed against any civilian population, with knowledge of the attack.			
Murder	*They (military) wrapped them in hay, poured fuel on them, and burned them to ash inside the fire of the burning houses. (Rathedaung #9)*	12% of hamlet leaders reported observing mass graves in their hamlet or en route to Bangladesh.	Physical exams were conducted among survivors; injuries were consistent with testimony of attempted murder by gunshot, explosive devices, and burning.
Extermination	*They set up landmines in the fields outside all the Rohingya villages… they took positions on the side of the road… they started to shoot with launchers inside the village and set fire to the houses. Then people ran out of the houses. When people ran out of the village and reached the field outside, around 100 people died in the field. (Buthidaung #17)*	Deaths from violent and non-violent causes during attacks and displacement produced a crude mortality rate of 8.7 per 1000 persons.	42% of physical exam participants reported that family members were dead or missing.
Enslavement	*They used to ask for 20–30 laborers daily for working at the (BGP/Police) camp. They make people work at the camp, like construction in the camp, clearing bushes, and other things. They don’t pay people even one penny. (Rathedaung #13)* *We were used as forced labors. They paid us nothing. When we asked for fees, they started torturing us, saying that it was the fees for us. (Buthidaung #4)*	(Not measured)	(Not reported)
Deportation or forcible transfer of population	*The Battalion Commander said “We have been sent here from Burma to kill and eliminate you Bengalis. We have been sent here to attack Bengalis, to burn the houses of Bengalis. You leave as early as possible. Otherwise, we will keep on burning down (your villages) one after one. We will drive you by shooting and by attacking you with launchers. You all leave.” (Rathedaung #5)*	An estimated 233,826 Rohingya were internally displaced or remained within the northern Rakhine state. After the attacks, an estimated 665,101 Rohingya people were displaced to and living in Bangladesh; 644 Rohingya people were estimated to be missing as a result of the August 2017 attacks.	All survivors had been displaced to Bangladesh.
Imprisonment or other severe deprivation of physical liberty in violation of fundamental rules of international law	*Out of 45 people arrested, one died due to beating. All the rest were taken away. Some of them were released later by taking ransom. People who could not afford to pay ransom were not released. They are still in the prison. (Maungdaw #36)*	74% of Rohingya leaders reported that Rohingya individuals in their hamlets had been arrested in the period between Ramadan (June 24, 2017) and their flight during the August attacks.	Two interviews were conducted with men who reported being taken from their villages by soldiers before August 26 and detained and tortured as part of ongoing military actions against the Rohingya. These men were part of a group of 67 men reportedly taken from their villages, detained, and tortured during interrogations about political or terrorist affiliations until they were able to pay for their own release.
Torture	*They dehumanized us in many ways. Life was always in fear for being arrested, tortured, and imprisoned. (Maungdaw #7)*	(Physical violence measured, but no measure of torture was included)	Two interviews were conducted with men who reported being taken from their villages by soldiers before August 26 and detained and tortured as part of ongoing military actions against the Rohingya. These men were part of a group of 67 men reportedly taken from their villages, detained, and tortured during interrogations about political or terrorist affiliations until they were able to pay for their own release.
Rape, sexual slavery, enforced prostitution, forced pregnancy, enforced sterilization, or any other form of sexual violence of comparable gravity	*They raped women/girls in 8–10 houses. There included young adolescent girls, married women, daughters of the family, daughters-in-law of the families. They gathered all girls and women at one place and raped these (who they found good-looking). (Maungdaw #36)* *They raped women and girls in front of parents and siblings. So, it is the worst of humanity. (Maungdaw #41)*	28% of hamlets reported sexual violence and rape during the attacks.	Survivors interviewed during physical exams reported rape, mass rape, and breast mutilation. Several reported being left to burn in buildings. Scars and burns were consistent with testimony.
Persecution against any identifiable group or collectivity on political, racial, national, ethnic, cultural, religious, gender as defined in paragraph 3, or other grounds that are universally recognized as impermissible under international law, in connection with any act referred to in this paragraph or any crime within the jurisdiction of the Court;	*They used to raid the villages often and trouble the villagers a lot. They used to threaten the villagers, beat the villagers, arrest them, and extort money from the villagers.* *The police usually patrolled inside the village at nights and if they found any small light inside the house, for example, while people were having a meal before sunrise during Ramadan, they used to grab people out of the house and beat them. (Buthidaung #9)* *They had been persecuting us such as we had to give them money continuously. If we don’t give money to them, they badly beat us, break the bones. (Buthidaung #12)*	In addition to widespread murder and violence against all Rohingya, 63% of hamlets reported that religious leaders had been targeted during the attacks and 69% reported that mosques had been burned or destroyed. Additionally, 92% of hamlets reported official meetings prior to the attacks; 94% of these were to inquire about NVCs and 81% of these meetings were perceived as a threat to move.	Survivors reported that mosques and crops and other forms of livelihood were often destroyed during the attacks.
Enforced disappearance of persons;	*Although there were no terrorists, the military arrested wise and wealthier people from the hamlet. (Maungdaw #12)* *Many people were arrested and taken away. We estimate around 65 people were arrested. Some of them went missing. We don’t know their whereabouts. (Rathedaung #2)*	An estimated 4605 Rohingya were arrested between Ramadan and the August 2017 attacks; of the 74% of hamlets in which members were arrested, 94% indicated that no reason was given for the individuals’ arrest.	(Not reported beyond arrests or missing persons)
Other inhumane acts of a similar character intentionally causing great suffering, or serious injury to body or to mental or physical health.	*Six kids were shot to death and put in a mass grave. After shooting them, the military sent their dogs to bite them. Soon, the military slit their throats and cut off their genitals and took out their eyes. It was heart-stopping seeing that. (Maungdaw #7)*	Common physical violence against Rohingya people included injury with weapons, gunshot wounds, tying people to or trapping them within burning structures, sexual assault and mass rape, and injury or death by mortars, landmines, RPGs, and grenades, including as Rohingya were fleeing their hamlets.	Physical findings included gunshot injuries, blunt trauma, penetrating trauma such as slashings and mutilations, burns and explosive injuries, and injuries from sexual and gender-based violence.

Findings provided in this report and others such as those by PHR [13], the United States Department of State [15], Amnesty International [12], the UN Human Rights Council [22], and Médecins Sans Frontières [14] are consistent with definitions of crimes against humanity and may constitute violations of the Genocide Convention. These crimes are not spontaneous or isolated events, but rather unfold on a large scale and as a systematic process, with precursors and triggering factors. The Convention on the Prevention and Punishment of the Crime of Genocide (commonly referred to as the “Genocide Convention”), which Myanmar ratified in March 1956, specifically criminalizes acts “committed with intent to destroy, in whole or in part, a national, ethnical, racial or religious group.” The founding treaty of the International Criminal Court (ICC), the Rome Statute, enumerates the following acts as crimes against humanity: murder, extermination, enslavement, deportation or forcible transfer of populations, imprisonment in violation of international law principles, torture, sexual violence, persecution of any identifiable group or collectivity, enforced disappearance, apartheid, and other inhumane acts intentionally causing great suffering or serious injury to body or to mental or physical health.

To determine whether acts constitute crimes against humanity, two main elements must be established: 1) that these acts were committed as part of a widespread and systematic attack directed against a civilian population; and 2) that the perpetrator participated in and had knowledge of the attack [25]. To analyze and assess the crimes committed against the Rohingya as potential atrocity crimes as defined by international law, the UN Human Rights Council established an independent fact-finding mission. The mission’s first report states that the Myanmar security forces committed serious crimes against humanity, including elements of extermination and deportation. It concludes: “there is sufficient information to warrant the investigation and prosecution of senior officials in the *Tatmadaw* chain of command, so that a competent court can determine their liability for genocide [22]”. Further exploration by the fact-finding mission into the economic interest of the Myanmar military at the time of the attacks, as detailed in its latest report [26], provides additional evidence. The mission identified businesses and organizations from which the military solicited and received donations totaling USD 10.2 million in support of their “clearance operation.”

Fortify Rights and the Simon-Skjodt Center for the Prevention of Genocide at the U.S. Holocaust Museum produced similar evidence demonstrating that Myanmar security forces and civilian perpetrators committed crimes against humanity [11]. In its 2018 report, Fortify Rights took this argument further, reporting that the following six strategies and behaviors employed by the Myanmar authorities amounted to genocide, in addition to crimes against humanity: 1) systematic collection of sharp/blunt objects from Rohingya civilians to “disarm” them; 2) training and arming of local non-Rohingya ethnic citizens in northern Rakhine state to prepare them for violence; 3) systematic destruction of fencing and other structures around Rohingya homes to provide attackers with a greater line-of-sight on civilians; 4) deprivation of Rohingya civilians of food and other lifesaving aids and systematic physical weakening of Rohingya ahead of attacks; 5) deployment of an unnecessarily high number of state security forces to northern Rakhine state; and 6) violations of human rights against Rohingya civilians, including imposing discriminatory curfews and other violations prior to attacks [24].

Findings of genocidal acts associated with the August 2017 attacks can be situated within a broader investigation of the decades-long mistreatment and persecution of the Rohingya people in Myanmar. In 2015, the International State Crime Initiative (ISCI) published a report that systematically reviewed State-led policies, laws, and strategies, ultimately concluding that these State-led efforts constituted a genocidal process against the Rohingya people. At the time of this report – even before the August 2017 attacks – the authors raised concerns that the Rohingya faced the “final two stages of genocide – mass annihilation and erasure of the group from Myanmar’s history [27]”. Thus, the results from this study and those of others situate the August 2017 attacks within this historical process of genocide of the Rohingya in Myanmar [15, 24, 28]. Community resilience, especially in conflict zones and in communities experiencing structural violence, has been a critical issue for research, humanitarian support, and public health [29]. Qualitative participants shared a narrative that explains past and present experiences and, for many, paints a common picture for their desired future. The process – a collective story of adversity – along with their shared cultural values constructs meaning and imparts a moral and social order in their lives. Our data presents poignant testimonies of everyday adversity and suffering as well as acute, horrific acts of violence. Qualitative participants spoke of hope for justice, hope for freedom of education and religion, hope for change and peace, hope for citizenship, and hope to return home to live in safety and with dignity.

As seen in many communities forced to endure everyday hardship and social disadvantage, adults will see hard work and education for the next generation as a gateway to economic security [30]. Their resilience is rooted in a strong conviction that their children will be well-educated. Qualitative participants echoed similar pleas for access to quality education given the years of interrupted schooling for their young and lost opportunities for their older youth. Currently, the government of Bangladesh does not permit Rohingya refugees to attend state-sponsored schools [31, 32]. Education provision must be improved to further goals of psychosocial wellbeing in the lives of the young Rohingya generation. Viewing the problem with a wider lens and integrating education into peacebuilding efforts at a global and country level is crucial.

Findings reported here should be viewed in light of study limitations. The actions by Myanmar state security forces documented in our qualitative findings may not capture the full extent of the human rights violations that were perpetrated against the Rohingya. For example, other reports have documented sexual violence perpetrated against Rohingya men and boys, as has been witnessed in other war settings [18, 33, 34]. As our qualitative interviews were conducted among male hamlet leaders from northern Rakhine state, it is likely that some survivors of sexual violence, including both female and male survivors, did not report their experiences to these leaders due to high levels of shame among survivors. While the inclusion of hamlet leaders, represented only by adult men, may introduce some limitations, we considered this to be the most ethically sound option that allowed for the collection of high quality, accurate data for the affected villages. Further, the mixed-methods nature of this research provides a comprehensive assessment of human rights violations and mortality during the August 2017 attacks. Each approach offers important strengths, and it is their simultaneous integration and synergy that provides a unique contribution to the study of measuring human rights violations as well as evidence of coordinated, widespread, and systematic attacks against the Rohingya population.

## Conclusion

These findings offer qualitative depth to our previously published assessment of the extent of the violence against the Rohingya as an ethnic group [9] and provide poignant insight into the persecution of the Rohingya leading up to and during the August 2017 attacks. The Rohingya have endured pervasive abuse and experienced atrocity crimes committed by Myanmar’s Border Guard Police, military, and neighboring ethnic Rakhine civilians, providing a narrative that is consistent with crimes against humanity and acts that may constitute violations of the Genocide Convention, as documented by other reports, such as those by PHR [13], the U.S. Department of State [15], Amnesty International [12], and MSF [14], among others.

Attacks on the Rohingya have not been adequately investigated and perpetrators have not been held accountable. The actions taken by the Myanmar government may be investigated per the Rome Statute of the ICC or before other ad hoc tribunals that have jurisdiction to try individuals for serious international crimes, including crimes against humanity and genocide [35]. Meanwhile, it is critical for the government of Myanmar to grant unfettered access to Rakhine state for independent monitors, international human rights organizations, journalists, aid agencies, medical professionals, and other international observers [13, 35]. Finally, any discussion around Rohingya repatriation must hinge on actionable guarantees and sustainable conditions for safe, dignified, and voluntary return, as described by study participants. This includes granting full citizenship and equal rights for the Rohingya.

At this time, and in light of subsequent attacks that have followed those perpetrated in August 2017 [28], gross impunity for the egregious human rights abuses perpetrated by security forces persists. As a country that voted in favor of the Universal Declaration of Human Rights, Myanmar must move toward embracing accountability, guaranteed legal protection for the rights of all its people – including the Rohingya and other ethnic minorities – and a zero tolerance approach to abuses.

## Data Availability

The datasets generated and/or analyzed during this assessment are not publicly available due to the risk of retaliatory persecution of study staff and respondents by state authorities. Anonymous data may be made available from the corresponding author, NM, upon reasonable request.

## Abbreviations

ARSA: Arakan Rohingya Salvation Army
BGP: Border Guard Police
ICC: International Criminal Court
NVC: National Verification Card
PHR: Physicians for Human Rights
UN: United Nations
UNHCR: United Nations High Commissioner for Refugees
